# Robotic radical prostatectomy: analysis of midterm pathologic and oncologic outcomes: A historical series from a high-volume center

**DOI:** 10.1007/s00464-020-08177-0

**Published:** 2020-12-07

**Authors:** Anastasios D. Asimakopoulos, Filippo Annino, Camille Mugnier, Laurent Lopez, Jean Luc Hoepffner, Richard Gaston, Thierry Piechaud

**Affiliations:** 1 Department of Urology, Clinique Saint Augustin, Bordeaux, France; 2 Department of Surgical Sciences, Unit of Urology, Fondazione PTV Policlinico Tor Vergata, Rome, Italy; 3 Unit of Urology, Ospedale San Donato, Arezzo, Italy

**Keywords:** Cancer of prostate, Robotics, Prostatectomy, Laparoscopy, Positive surgical margin

## Abstract

**Background:**

Identifying predictors of positive surgical margins (PSM) and biochemical recurrence (BCR) after radical prostatectomy (RP) may assist clinicians in formulating prognosis. Aim of the study was to report the midterm oncologic outcomes, to identify the risk factors for PSM and BCR and assess the impact of the PSM on BCR-free survival following robot-assisted laparoscopic radical prostatectomy (RALP).

**Methods:**

From 2005 to 2010, 1679 consecutive patients underwent transperitoneal RALP. Data was retrospectively collected by an independent statistical company and analyzed in 2014. Median postoperative follow-up was 33.5 mo. BCR was defined as any detectable serum prostate-specific antigen (PSA) ≥ 0.2 ng/mL in two consecutive measurements. BCR-free survival was estimated using the Kaplan–Meier method. Univariate and multivariate analysis were applied to identify risk factors for PSM and BCR.

**Results:**

In pN0/pNx cancers, pathologic stage was pT2 in 1186 patients (71.8%), pT3 in 455 patients (27.6%), and pT4 in 11 patients (0.6%). PSM rate was 17.4% and 36.9% of pT2 and pT3 cancers, respectively. Pathologic Gleason score was < 7, = 7 and > 7 in 42.1%, 53% and 4.9% of the patients, respectively. Overall BCR-free survival was 73.1% at 5 years; the 5-year BCR-free survival was 87.9% for pT2 with negative surgical margins. PSA, Gleason score (both bioptic and pathologic), pathologic stage (pT) and surgeon's volume were significant independent predictors of PSM. PSA, pathologic Gleason score, pT and PSM were significant independent predictors of BCR-free survival. Seminal vesicle-sparing, nerve-sparing approach and the extent of nerve-sparing (intra vs interfascial dissection) did not negatively affect margin status or BCR rates.

**Conclusions:**

PSMs are a predictor of BCR. Being the only modifiable factor influencing the PSM rate, surgical experience is confirmed as a key factor for high-quality oncologic outcomes.

Despite the current, conflicting evidence regarding the benefit of radical prostatectomy (RP) over deferred treatment in both the overall and cancer-specific survival of patients affected by localized prostate cancer (PCa), RP still remains a mainstay of treatment [1]. Robot-assisted laparoscopic RP (RALP) has become the established state-of-the-art surgical treatment for PCa [2].

Although many patients are disease-free after surgery, nearly 30% [3] of patients experience biochemical recurrence (BCR). Defined as a detectable prostate-specific antigen (PSA) level following RP in the absence of clinical progression, BCR is the most common pattern of disease relapse [4]. Patients with BCR have a considerably worse prognosis, often develop metastasis, and can die of the disease [3, 4]. Therefore, identifying prognostic predictors of BCR after RP to assist clinicians in predicting outcomes for decision-making is required. Moreover, although positive surgical margin (PSM) is frequently reported in RP series, their clinical relevance remains uncertain despite extensive investigation. Several studies demonstrated an association between PSM and BCR [5–7], while others have observed insignificant or even contrary correlations [8–10].

Aim of this study was to report the midterm oncologic outcomes, to identify the risk factors for PSM and BCR and assess the impact of the PSM on BCR-free survival following RALP.

## Materials and methods

From January 2005 to June 2010 1679 consecutive patients underwent RALP for localized PCa at our department. All data was retrospectively collected into a customized database and analyzed by an independent statistical company in 2014.

All RALP were performed transperitoneally with an antegrade approach through the Retzius space by four laparoscopic urologists. No frozen sections were routinely obtained. When indicated, a nerve-sparing procedure and/or standard lymph node dissection was performed. In patients who did not undergo lymph node dissection, cancer was classified as pNx. The degree of nerve-sparing was decided on the basis of preoperative variables (PSA level, clinical stage, Gleason score, % and location of positive biopsies, % of involvement of the single bioptic cores, preoperative potency status as well as data of the magnetic resonance of the prostate –when available-) and intraoperative findings (visual cues such as changes in color or texture of the tissue, capsular flaps, bulging and surface irregularities, adhesiveness of planes or presence of the mass effect produced by the tumor). Periprostatic arteries or veins were not used as landmarks for the dissection.

History of previous abdominal, pelvic or prostatic surgery were not contraindications for RALP. Patients who had received neoadjuvant therapy or adjuvant therapy before PSA relapse were excluded from analyses.

The study was conducted in accordance with Good Clinical Practice rules and with the ethical principles contained in the Declaration of Helsinki as amended in Hong Kong. Each patient gave written informed consent, while the study protocol obtained regulatory ethical committee notification (PTV trials register 138.10). Baseline demographic and clinical characteristics of the patients (age, BMI, preoperative PSA, prostate volume, etc.) as well as all medical and surgical complications occurring both in inpatient and outpatient settings were recorded [11]. Prostatectomy specimens were analyzed for weight, pathologic stage, Gleason's grade, tumor location, margin status (positivity, location and extension).

The methods of processing the specimen have been previously described [12]. Briefly, the RALP specimens were cut into 5 mm axial sections, formalin-fixed and routinely processed for paraffin embedding. Subsequently the embedded specimens were cut into 5 μm sections and stained with haematoxylin and eosin. A PSM was reported if cancer cells were found at the inked specimen margin.

PSA data were collected every 4 months in the first year, then every 6 months for two years and then yearly. BCR was defined as two consecutive PSA rises ≥ 0.2 ng/ml [13]. The BCR-free survival was estimated using the Kaplan–Meier method. Survival curves were stratified by PSA level, pathologic features, surgical margins status, pathological stage and pathological stage/surgical margins. Pathologic Gleason score was divided as follows: Gleason score < 7, = 7, or > 7. PSA level was considered a qualitative variable as follows: PSA < 10 ng/ml or ≥ 10 ng/ml. The curves were compared using the log-rank test.

The risk factors for PSM (prevalence, localization, extension), BCR, early (within 2 years) or late BCR (after 2 years following RALP) were tested using univariate analysis and subsequently confirmed using a logistic regression model.

Young men with several risk factors for clinical failure (high pathologic Gleason score, multiple or extended PSM) were submitted to adjuvant radiotherapy. Instead, patients > 70 years or with an isolated focal PSM and otherwise low-risk organ-confined PCa close monitoring with serial PSA measurements was recommended, with radiotherapy at the earliest sign of recurring disease, if any.

A double-sided *p* value < 0.05 was considered statistically significant. All data were analyzed using SAS V9.3.

## Results

Median postoperative follow-up was 33.5 mo (Q1:Q3 = 12.1:54.2). Mean age was 61.5 years (SD 6.4) and mean preoperative PSA 7.37 ng/ml (SD 3.76). The majority of the patients (63%) were affected by T1c PCa, mainly of Gleason score 6 (3 + 3) (75%). 37% of the patients had received previous abdominal surgery and 2.7% previous prostatic surgery. Hypertension was the main comorbidity (29% of the patients). The baseline characteristics of the patients are summarized in Table 1.

**Table 1:** Demographic and preoperative clinical data and major comorbidities

Variable	Outcome	Number of patients with available data (%)
Number of patients	1679	
Mean age, years (SD)	61.5 (6.4)	1675 (99.8%)
Mean preoperative PSA, ng/ml (SD)	7.37 (3.76)	1322 (78.74%)
Clinical Gleason score, mean (SD)	6.2 (0.6)	1365 (81.3%)
Prevalence of clinical Gleason score (%)		1365 (81.3%)
≤ 6	75	
7	21.9	
8–10	3.1	
Clinical stage (%)		1360 (81%)
cT1	63	
cT2	31	
cT3	6	
Major comorbidities (%)		
Morbid obesity	0.4	1659 (98.8%)
Hypertension	29.2	1665 (99.2%)
Diabetes mellitus	2.5	1651 (98.3%)
Coronary artery disease	5.1	1658 (98.7%)
Myocardial infarction	1.7	1648 (98.2%)
Chronic kidney failure	0.2	1659 (98.8%)
Peripheral vascular disease	3.6	1654 (98.5%)
Mean preoperative hemoglobin, gr/dl (SD)	15.1 (1.1)	1360 (81%)
Previous abdominal surgery, overall (%)	37.1	1653 (98.5%)
Main previous surgical procedures		
Appendectomy (%)	53.4	
Inguinal hernia repair (%)	24.6	
Colon resection (%)	2.3	
Other (%)	19.7	

Number of evaluated patients for each variable is presented. *PSA* prostate-specific antigen, *SD* standard deviation

Mean operative time was 221 (SD 55.8) minutes, while mean console time was 117 min (SD 43.5). In the majority of the cases (89.2%) an interfascial dissection of the periprostatic neural network was performed. In the 33.9% of the cases a seminal vesicle-sparing approach was performed, while a running suture was mainly adopted for the vesico-urethral anastomosis with no posterior reconstruction of the Denonvillier’s fascia. Operative time, type of dissection, estimated blood loss, hospital stay, length of catheterization as well as the major intra and perioperative complications are summarized in Table 2.

**Table 2:** Intra and perioperative data

Variable	Outcome	Number of patients with available data (%)
Mean operative time (OR occupancy, minutes) (SD)	221.2 (55.8)	1633 (97.26%)
Mean console time, minutes (SD)	117.6 (43.5)	1543 (91.9%)
Mean skin to skin time, minutes (SD)	148.8 (43.6)	1600 (95.3%)
Intrafascial dissection	10.1%	1649 (98.2%)
Interfascial dissection	89.2%	
Extrafascial dissection	0.7%	
Seminal vesicle-sparing	33.9%	1667 (99.3%)
Running urethrovesical anastomosis	97.5%	1639 (97.6%)
Lymphadenectomy	132 pts	
Intraoperative complications		1679 (100%)
Severe bleeding	0.4	
Rectal injury *(%)	0.1	
Conversion to pure laparoscopy (%)	0.5	
Robot failure (%)	0.6	
Perioperative complications		
Hemorrhage**(%)	1.3	
Clot retention-bladder tamponade***(%)	2.2	
Anastomotic urine leak****(%)	0.2	
Blood transfusion (%)	3.5	1647 (98.1%)
Febrile urinary tract infection (%)	15	1679 (100%)
Ileal lesion (%)	0.1	1679 (100%)
Obstructive ileum*****(%)	0.2	1679 (100%)
Mean hospital stay, days (SD)	5.4 (2.6)	1659 (98.8%
Mean catheterization time, days (SD)	7.5 (2.6)	1234 (73.5%)

Actual number of patients with available data is shown. *OR* operating room, *SD* standard deviation

Intraoperative complications were managed as follows:

*Intraoperative suture without further sequelae

**Laparoscopic reintervention and assessment of the haemostasis

***Endoscopic clot evacuation and bladder irrigation

****Prolonged catheterization

*****Open abdominal surgery

Pathologic data and main oncologic outcomes are summarized in Table 3. Mean prostate weight was 44.4 ± 18.7 gr. In pN0/pNx cancers, postoperative stage was pT2a in 128 patients (7.8%), pT2b in 21 patients (1.3%), pT2c in 1037 patients (63.2%), pT3a in 357 patients (21.7%), pT3b in 98 patients (6%) and pT4 in 1 patient (0.1%). Pathologic Gleason score was 3 + 3 in 42.1% and 3 + 4 in 42.4% of the patients. In 12.3% of the patients a capsular incision was identified in the pathologic examination.

**Table 3:** Oncologic outcomes

Variable	Outcome	Number of patients with available data (%)
Pathologic stage (%)		1642 (97.8%)
pT2a	7.8	
pT2b	1.3	
pT2c	63.2	
pT3a	21.7	
pT3b	6	
pT4	0.1	
Capsule violation %	12.3	1612 (96%)
Mean prostate weight, gr (SD)	44.4 (18.7)	1654 (98.5%)
Mean pathologic Gleason score (SD)%	6.64 (0.63)	1652 (98.4%)
≤ 6	42.1	
7	53	
8–10	4.9	
Positive surgical margins %*	22.6	1657 (98.7%)
PSM/pT%		
pT2	17.4	
pT3	36.9	
Biochemical recurrence %**	15.5	1312 (78.1%)
Secondary treatment (adjuvant/salvage RT or HT) %**	23.8	1364 (81.2%)

Actual number of patients with available data is shown. *SD* standard deviation, *gr* grams, *PSM* positive surgical margins, *RT* radiotherapy, *HT* hormone-therapy

Overall PSM rate was 375/1657 (22.6%). Margin rate per stage was 17.4% and 36.9% of pT2 and pT3 cancers, respectively. PSM were mainly localized at the level of the apex with a similar distribution for both lobes as shown in Table 4.

**Table 4:** Overall distribution of the positive surgical margins (PSM) per lobe

PSM location	Left	Right
Apex focal	84	95
Apex extensive	18	16
Posterolateral focal	36	28
Posterolateral extensive	10	9
Base focal	29	27
Base extensive	7	11
Bladder neck focal	1	2
Bladder neck extensive	1	1
Overall	186	189
	375/1657 (missing values: 22)	

Length of PSM was defined as focal or extensive. Focal PSM: single PSM (sPSM) ≤ 3 mm;—Extensive PSM: sPSM with linear length > 3 mm or several margins regardless of the length

**Fig. 1:**
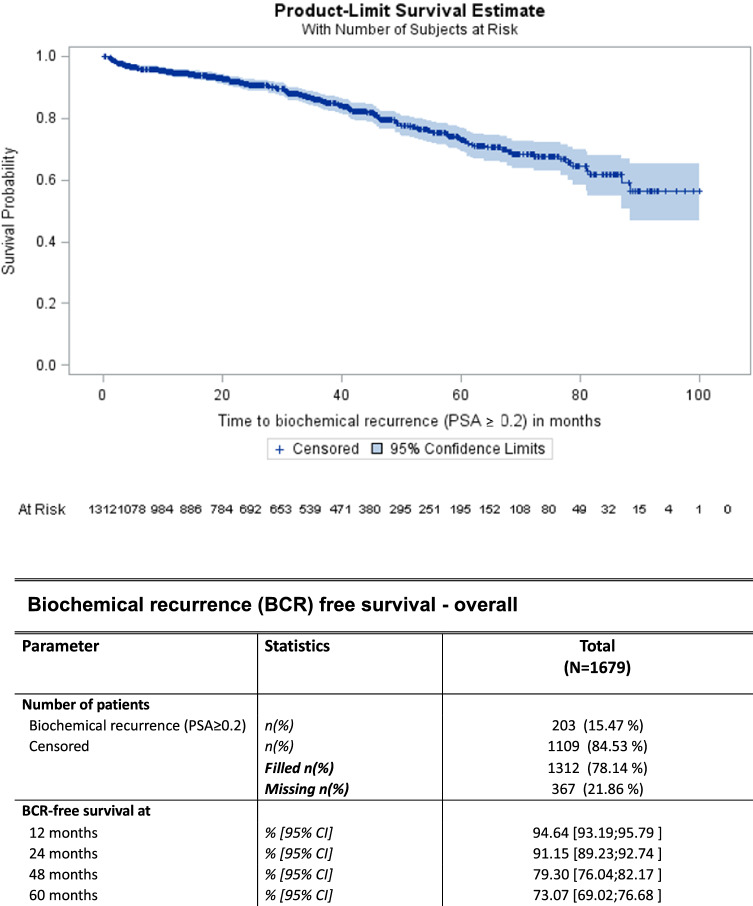
Biochemical progression-free survival. 367 observations with invalid time or censoring values were deleted

1312/1679 patients (78.1%) received at least one follow-up visit. 203/1312 patients experienced BCR during follow-up (15.5%). The BCR-free survival (BCRFS) at 12, 24, 48 and 60 months after RALP were 94.6%, 91.2%, 79.3% and 73.1%, respectively (Fig. 1).

**Fig. 2:**
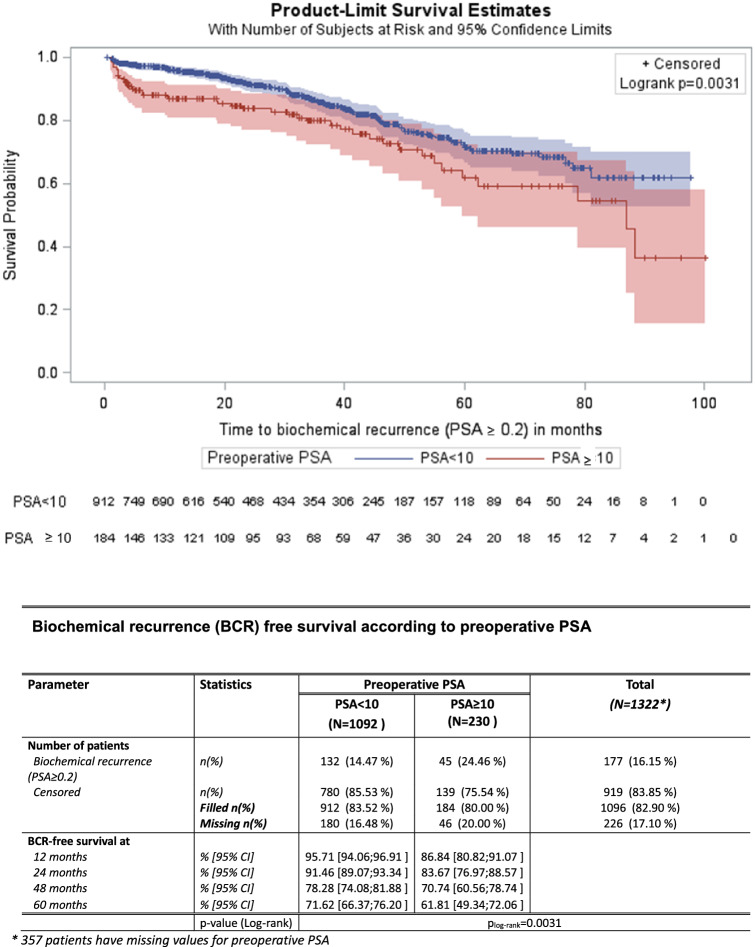
Biochemical progression-free survival according to preoperative PSA

The 5-year BCRFS was 71.6% for PSA < 10 ng/ml and 61.8% for a PSA > 10 ng/ml. The relative survival curves were statistically different (Fig. 2, *p* = 0.003).

**Fig. 3:**
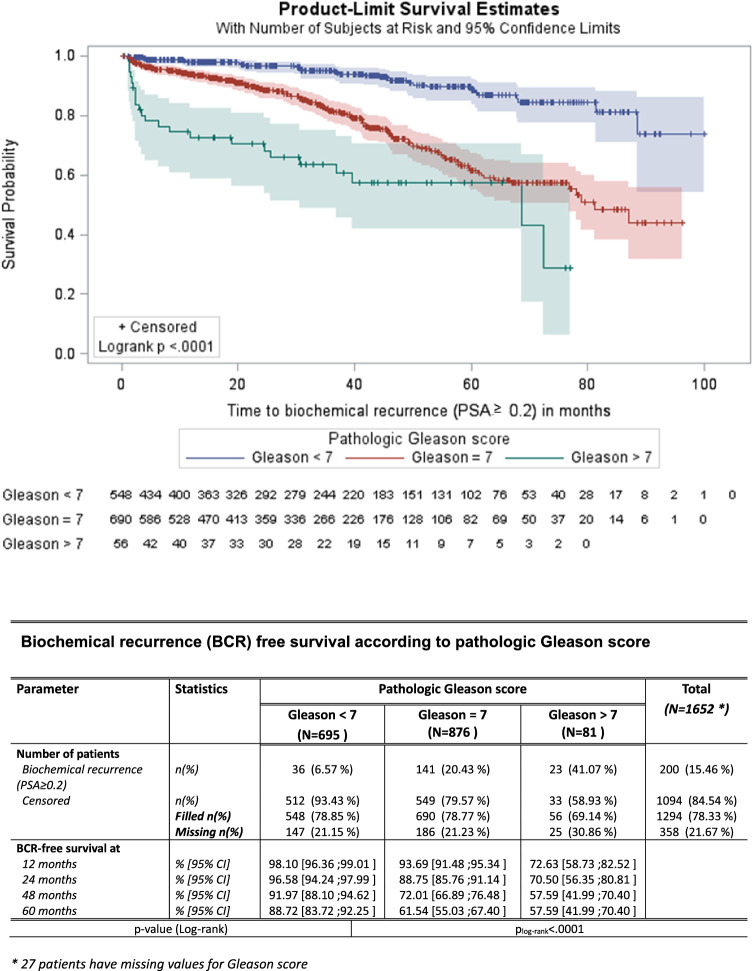
Biochemical progression-free survival according to pathologic Gleason score

According to Gleason score, difference between survival curves also reached significance (*p* < 0.0001). Patients with a Gleason score < 7 had a 5-year BCRFS of 88.7% compared with 61.5% for those with Gleason score = 7 and with 57.6% of those with Gleason score > 7 (Fig. 3).

**Fig. 4:**
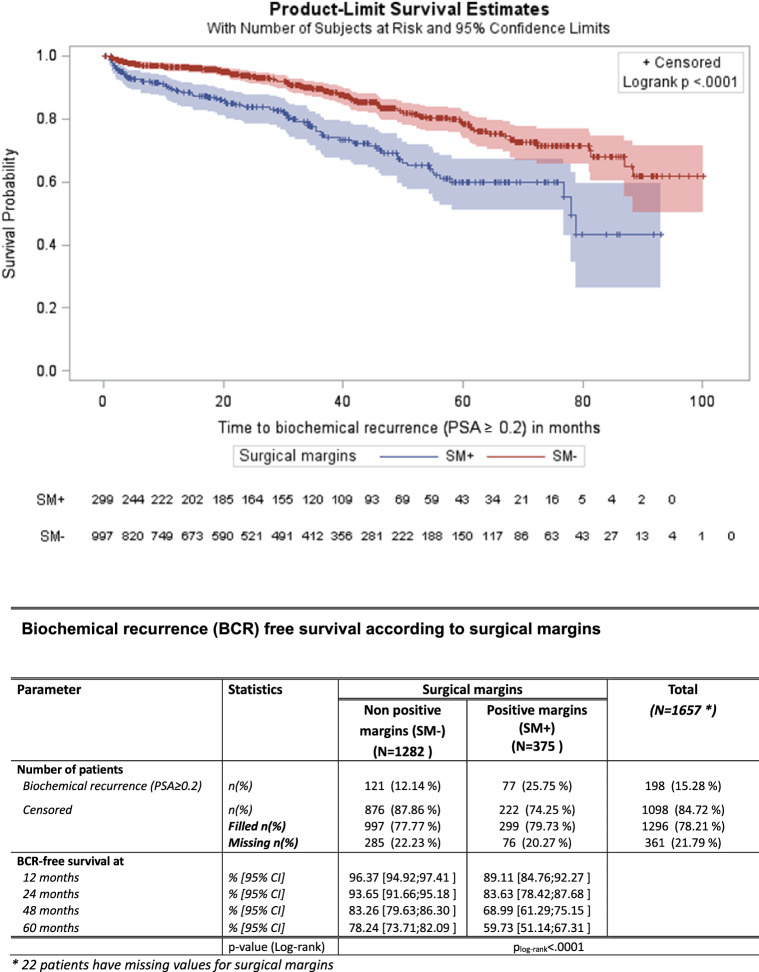
Biochemical progression-free survival according to surgical margins. SM +  = positive surgical margins, SM − = negative surgical margins

Surgical margin status was a predictor of PSA recurrence by the log-rank test (Fig. 4). 5-year BCRFS for patients with negative surgical margins was 78.2% compared to 59.7% for patients with PSM (*p* < 0.0001).

**Fig. 5:**
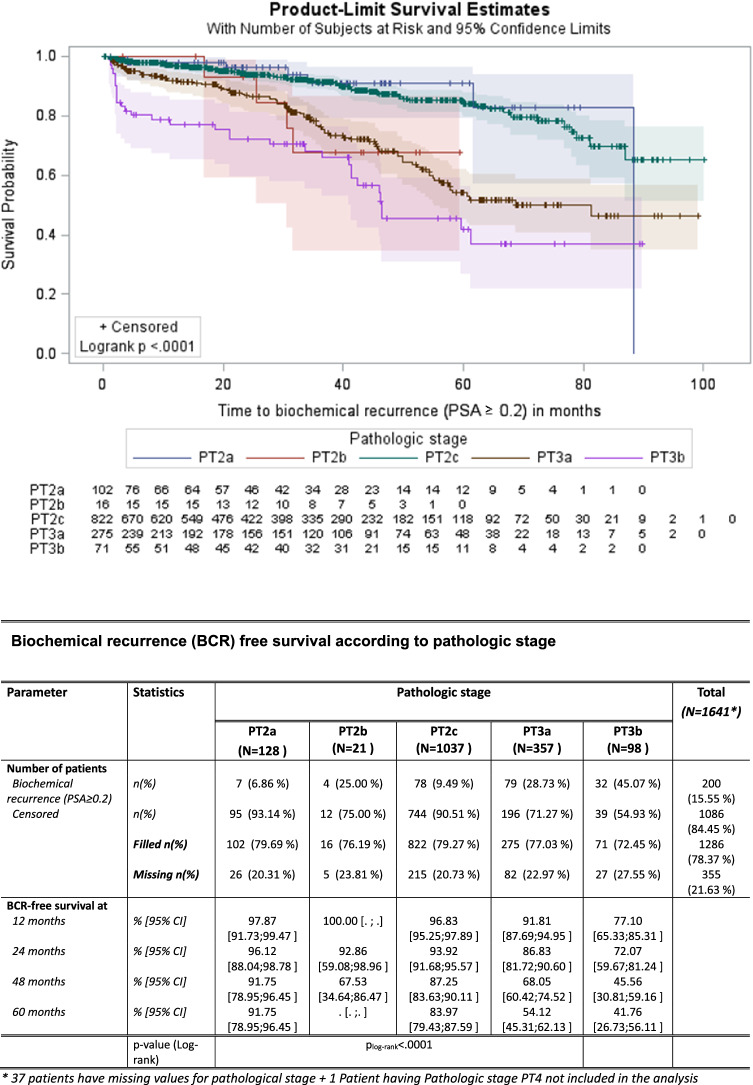
Biochemical progression-free survival according to pathologic stage

According to the pathologic stage, 5-year BCRFS was 84% for pT2c vs 54.1% for pT3a and 41.8% for pT3b (*p* < 0.0001). Pathologic stage was a predictor of PSA recurrence by the log-rank test (Fig. 5).

**Fig. 6:**
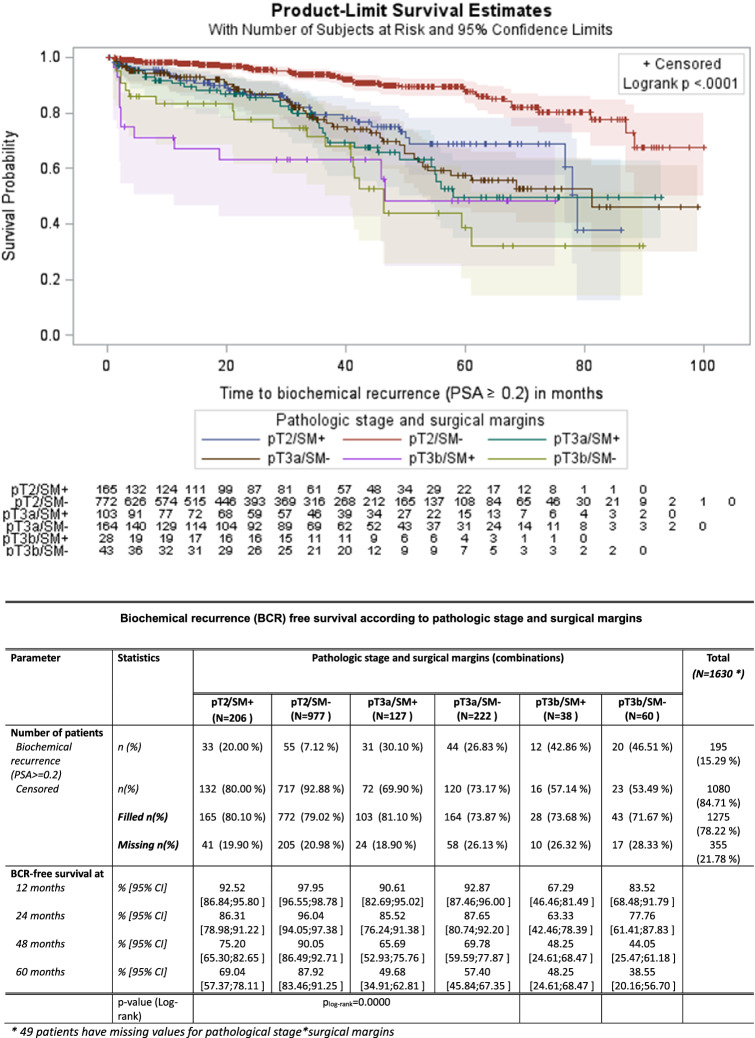
Biochemical progression-free survival according to pathologic stage and surgical margins

When analysis was stratified by pathologic stage and margin status, the 5-year BCR-free survival rate was 87.9% for pT2 with negative surgical margins. Cases of pT2 with PSM and pT3a with negative surgical margins were characterized by similar 5-yr progression-free survivals rates (69% and 57.4%, respectively, p = 0.34) (Fig. 6).

At the univariate analysis (chi^2^ or t-Student), preoperative PSA ≥ 10 (*p* = 0.01), preoperative Gleason score (*p* = 0.02), lower specimen weight (mean non-PSM 45.7 ± 18.93, mean PSM 39.8 ± 16.21, *p* < 0.0001), higher pathologic stage or pathologic Gleason score and lower surgeon volume were the possible predictive factors of PSMs, while the previous prostatic or abdominal surgery, the type of NVB dissection (extra vs intra vs interfascial) and the conservation of the tip of the seminal vesicles were not associated with PSM (Table 5). At the multivariate analysis the preoperative PSA ≥ 10 ng/ml, the pT3 stage, the preoperative Gleason score > 7 or the pathologic Gleason score ≥ 7 as well as a surgeon's volume < 100 cases per year were confirmed as predictive factors for PSM (Table 6). No factor was identified in the univariate analysis that could predict the location or the extent of the PSM.

**Table 5:** Univariate analysis for different variables for prediction of positive surgical margins

	PSM	Negative SM	p
Prostate weight (n, mean, sd)	368, 39.7 (16.2)	1273, 45.7 (18.9)	< 0.0001*
Preoperative PSA n (%)			0.01^
< 10 ng/ml	230 (21.4)	847 (78.6)	
> = 10 ng/ml	66 (29.1)	161 (70.9)	
Preoperative Gleason score n (%)			0.02^
< 7	216 (21.4)	793 (78.6)	
= 7	87 (29.4)	209 (70.6)	
> 7	7 (16.3)	36 (83.7)	
Previous Prostate surgery n(%)			0.76^
No	359 (22.9)	1210 (77.1)	
Yes	9 (20.9)	34 (79.1)	
Previous abdominal surgery n(%)			0.64^
No	237 (23.1)	788 (76.9)	
Yes	134 (22.1)	472 (77.9)	
Pathologic stage n(%)			< 0.0001^
pT2a	12 (9.4)	116 (90.6)	
pT2b	2 (9.5)	19 (90.5)	
pT2c	192 (18.6)	842 (81.4)	
pT3a	127 (36.4)	222 (63.6)	
pT3b	38 (38.7)	60 (61.3)	
Pathologic Gleason score n (%)			< 0.0001^
< 7	96 (13.9)	593 (86.1)	
= 7	247 (28.3)	625 (71.7)	
> 7	28 (35)	52 (65)	
Type of neurovascular tissue dissection n(%)			0.68^
Extra	1 (11.1)	8 (88.9)	
Inter	258 (22.5)	887 (77.5)	
Intra	31 (23.7)	100 (76.3)	
Conservation of the tip of the seminal vesicles n(%)			0.35^
No	254 (23.4)	833 (76.6)	
Yes	119 (21.3)	439 (78.7)	
Surgeon’s volume n(%)			0.02^
> 200/year	187 (21.8)	672 (78.2)	
100–200/year	70 (19.1)	296 (80.9)	
< 100/year	118 (27.3)	314 (72.7)	

Variables with a p value < 0.10 at the univariable analysis, were considered in the multivariable model. *PSM* positive surgical margins, *SM* surgical margins, *PSA* prostate-specific antigen

*T test; ^Chi-square or Fisher exact test

**Table 6:** Multivariate Cox proportional hazard model for different variables for prediction of positive surgical margins

Variable	Risk ratio	95% CI	P value
PSA < 10 ng/ml	1		
PSA ≥ 10 ng/ml	1.48	1–2.17	0.04
Gleason score < 7	1		
Gleason score = 7	1.28	0.52–1.80	0.27
Gleason score > 7	1.78	0.84–1.93	0.03
Pathologic stage pT2a	1		
pT3a	3.14	1.52–6.48	0.002
pT3b	2.83	1.2–6.68	0.01
Pathologic Gleason score < 7	1		
= 7	1.45	0.85–2.46	0.17
> 7	1.84	1.24–2.48	0.001
Surgeon’s volume > 200 cases/year	1		
< 100	1.41	1.01–1.98	0.04
100–200	0.70	0.49–1.03	0.07

Factors predicting biochemical recurrence in the univariate model were the presence and location of the positive surgical margins, the pathologic stage and pathologic Gleason score, the non-seminal vesicle-sparing technique and the infraction of the prostate capsule during surgery (Table 7). The multivariate analysis confirmed that only the PSM and the higher pathologic stage and Gleason score were predictive of BCR (Table 8), while the seminal vesicle-sparing does not increase BCR rates. No factor predictive of an early (≤ 2 years) versus late (> 2 years) BCR at univariate analysis was confirmed at the multivariate analysis.

**Table 7:** Univariate analysis for different variables for prediction of biochemical recurrence

	No BCR		BCR		p^
	n	%	N	%	
Positive surgical margins					< 0.0001
No	750	76.5	230	23.5	
Yes	167	56.4	129	43.6	
Pathologic stage					< 0.0001
pT2a	83	83	17	17	
pT2b	12	75	4	25	
pT2c	640	79.1	169	20.9	
pT3a	149	54.8	123	45.2	
pT3b	25	35.7	45	64.3	
Pathologic Gleason score					< 0.0001
< 7	426	79.9	107	20.1	
= 7	463	67.6	222	32.4	
> 7	22	39.3	34	60.7	
Prostate capsule infraction					< 0.0001
No	805	73.9	284	26.1	
Yes	87	56.9	66	43.1	
Conservation of the tip of the seminal vesicles					< 0.0001
No	635	68.6	291	31.4	
Yes	284	79.6	73	20.4	
Type of neurovascular bundle dissection					0.88
Extra	3	75	1	25	
Inter	604	69	271	31	
Intra	81	71	33	29	

*BCR* biochemical recurrence

^Chi-square test

**Table 8:** Multivariate Cox proportional hazard model for different variables for prediction of biochemical recurrence

Variable	Risk ratio	95% CI	P value
Negative surgical margins	1		
Positive surgical margins	1.86	1.35–2.58	0.0001
Pathologic stage pT2a	1		
pT3a	2.86	1.51–5-43	0.0012
pT3b	4.69	2.03–10.85	0.0003
Pathologic Gleason score < 7	1		
= 7	2.05	1.29–3.25	0.002
> 7	3.3	1.67–6.55	0.0006
Conservation of the tip of the seminal vesicles	1		
No conservation	1.59	1.15–2.18	0.004
Capsule infraction	1		
Non-capsular infraction	0.91	0.6–1.39	0.67

324/1364 patients (23.8%) received at least one secondary treatment among radiotherapy (210/324, 64.8%), hormonal treatment (154/324, 47.5%) and chemotherapy (1/324, 0.3%).

## Discussion

Identifying factors that predict the incidence of PSM and/or BCR may help physicians to adequately inform patients who are more likely to receive adjuvant multimodal therapy following RP.

PSMs after RP are generally considered an adverse outcome associated with failure of the surgery to achieve cure of PCa, tumor recurrences and debilitating additional therapies [14]. In the current era of the RALP, the prevalence of the PSMs ranges between 6.5% and 32%, with a mean value of 15% [15]. The distribution of the PSMs per pathologic stage varies between series, with a mean PSM rate of 9% in pT2 cancer (range 4–23%), 37% in pT3 (29–50%) and 50% in pT4 cancers (40–75%) [15]. In our study, although regarding the outcomes of a historical series, both the overall PSM rate (22.6%) and the PSM/pT rate was conform to the published literature.

Several studies identified clinical and pathologic factors predicting PSM most of which cannot be altered by the treating physician. Ficarra et al. [16] reported that prostate volume and cT stage were the only clinical variables predictive of any PSM, whereas pT stage was the unique pathologic predictor. In another study, patient's body mass index (BMI), PSA level, pT stage and prostate volumes (all *p* values < 0.001) were independent predictors of any PSM, whereas Gleason score was not. Similarly, BMI, PSA and prostate volume were predictors of PSMs in pT2 cancers [17]. In our series, multivariate Cox model showed that PSA, Gleason score (both bioptic and pathologic), pathologic stage (pT) and surgeon's volume below 100 cases/year were significant independent predictors of PSM. In the univariate analysis no factor that could predict the location or the extent of the PSM was identified.

Regarding BCR rates Badani et al., in the first study of large-scale oncological outcomes after RALP, reported a BCR rate of 7.3% in 2766 patients undergoing RALP at a median follow-up of 22 months. In that study, no detailed analysis of predictors of BCR was undertaken [18]. Menon et al. reported a 13.6% probability of BCR at 5 years for 1384 patients submitted to RALP [19]. Sukumar S et al. report a 81% of BCR-free survival at 8 years after RALP [20]. Murphy et al. reported on 400 patients with a BCR-free survival of 87% at a median follow-up of 22 months [21] while Suardi et al. reported 3- and 5-year BCR-free survival rates of 94% and 86% in 184 patients with a minimum follow-up of 5 years [22]. Sooriakumaran et al. reported a BCR-free survival rate of 84.8% at a median follow-up of 6.3 years [7], while Liss et al. reported a 84.9% at 5 years [23]. More recently, Diaz M et al. reported 10-year oncologic data on 483 patients that were submitted to RALP; 10-year BCR-free survival was 73.1% [24].

In our study 15.5% of patients experienced BCR during follow-up. The BCR-free survival (BCRFS) at 12, 24, 48 and 60 months after RALP were 94.6%, 91.2%, 79.3% and 73.1%, respectively. The multivariate analysis confirmed that the locally-advanced disease (pT3 stage), the pathologic Gleason score ≥ 4 + 3 and the PSM were predictive of BCR. The first two factors are immutable and are manifestations of the biology of the disease; the last one is partially influenced by the surgeon's volume, highlighting the role of the surgeon who, as an independent predictor of outcome, can affect the prognosis ensuring better local control. Although the effects of PSM on the risk of BCR are still unclear in the published literature, our study suggests that PSM represent an independent risk factor for BCR and as shown, their rate is function of the surgeon’s experience. Location of the margins, pathologic stage and extent of dissection of the periprostatic neuronal network were not predictive of BCR. No factor predictive of an early (≤ 2 years) versus late (> 2 years) BCR at univariate analysis was identified at the multivariate analysis.

Other recent studies assessed the relationship between surgical experience and oncologic outcomes of RALP, demonstrating that greater surgeon experience was associated with a lower probability of PSM [25]. Being the only modifiable factor influencing the PSM rate in our study, surgical experience is confirmed as a key factor for high-quality oncologic outcomes. The low rate of the PSM of this historical series that conforms to the most recent RALP series suggests that previous surgeon’s experience, even with different surgical approaches to RP (open, laparoscopic) may also be at the basis of high-quality oncologic outcomes.

Institutions with smaller caseloads and more than one surgeon using the robotic device will possibly have more problems with the learning curve saturation. These centers may probably benefit of the current possibilities for intraoperative margin assessment [14]. Others suggest even the development of radical prostatectomy-only centers [26].

In fact, the lack of tactile sensation in robotic surgery leads surgeons to rely on compensatory visual strategies to overcome this handicap. As a result surgical experience, adoption of energy and tension-free dissection of the periprostatic neural network and enhanced vision in a bloodless field can compensate for the lack of tactile sensation allowing surgeons to identify correctly alarming visual cues [27] and adapt dissection of the NVT to the actual risk of PSM.

In our technique, the accurate dissection of the neurovascular triangle as a first step of the surgery identifies all the important structures that will guide the subsequent neurovascular dissection, such as the base of the prostate, the lateral aspect of the bladder neck, the neurovascular tissue, the Denonvillier’s fascia and the seminal vesicle. By this approach, a correct identification of the periprostatic fascial layers is obtained [12].

The dissection proceeds tension-free (in order to avoid creation of capsular flaps) and energy-free (in order to maintain the original color and texture of the tissues). Small arteries that leave the bundle to enter the prostate are gradually identified, clipped, and divided since traction on them could cause disruption of the capsule. The bloodless planes that separate without resistance are followed and developed; we prefer to cut sharply through veins and periprostatic tissue rather than forcing a blunt dissection that is more likely to produce a capsular flap exposing to higher risk of PSM.

It finally should be underlined that seminal vesicle-sparing did not negatively affect margin status or BCR rates, as also suggested by others [28], while nor the nerve-sparing neither the grading of nerve-sparing (intra vs interfascial dissection) was associated with worse cancer outcomes both in terms of PSM or BCR, as also suggested by two recent meta-analyses [29, 30].

Our outcomes -characterizing an era without wide adoption of magnetic resonance imaging for preoperative staging- probably show that selective nerve-sparing during RARP, using only the preoperative clinical variables and surgeon's intraoperative perception seems to provide reasonable intermediate term oncologic outcomes with acceptable PSM rate.

The major limitation of our study is its retrospective design. However, an independent statistical service company acquired and processed the data, avoiding potential biases of self-processing. The study population encompass the effects of periods of transition from laparoscopy to robotics, of experimentation with the new technique, of transfer of knowledge among the team members, and finally of progressive maturation. The effect of tumor location and tumor volume/maximum tumor diameter on the incidence of PSM/BCR was not evaluated. Concerning the importance of the case-volume of the surgeon on the outcomes, the study does not probably reflect the real-life, since the comparisons are performed between experts in prostatectomy and the observed differences, although statistically significant, are probably lower than the expected. However, it seems that even between experts in prostatectomy, higher surgical volume is associated to better outcomes. Finally, although oncologic outcome is best defined by cancer-specific survival, meeting such endpoint in prostate cancer requires significantly longer follow-up than that in the present study. However, the statistical analysis was performed in 2014, and consequently long-term outcomes including metastasis-free survival and overall survival are not available.

## Conclusions

PSMs after RP are a predictor of biochemical recurrence and they depend on surgeon’s experience. The low overall PSM and BCR rates reported in the current historical series of RALP reflects the importance of the previous surgeon’s experience even with different approaches in RP (open/laparoscopic) in obtaining high-quality oncologic outcomes.

## References

[1] Hamdy FC, Donovan JL, Lane JA, Mason M, Metcalfe C, Holding P, Davis M, Peters TJ, Turner EL, Martin RM, Oxley J, Robinson M, Staffurth J, Walsh E, Bollina P, Catto J, Doble A, Doherty A, Gillatt D, Kockelbergh R, Kynaston H, Paul A, Powell P, Prescott S, Rosario DJ, Rowe E, Neal DE (2016). ProtecT Study Group 10-Year Outcomes after Monitoring, Surgery, or Radiotherapy for Localized Prostate Cancer. N Engl J Med.

[2] Takenaka A, Ak T (2012). Anatomical basis for carrying out a state-of-the-art radical prostatectomy. Int J Urol.

[3] Isbarn H, Wanner M, Salomon G, Steuber T, Schlomm T, Köllermann J, Sauter G, Haese A, Heinzer H, Huland H, Graefen M (2010). Long-term data on the survival of patients with prostate cancer treated with radical prostatectomy in the prostate-specific antigen era. BJU Int.

[4] Pisansky TM, Thompson IM, Valicenti RK, D'Amico AV, Selvarajah S (2019). Adjuvant and Salvage Radiotherapy after Prostatectomy: ASTRO/AUA Guideline Amendment 2018–2019. J Urol.

[5] Hashimoto T, Yoshioka K, Horiguchi Y, Inoue R, Yoshio O, Nakashima J, Tachibana M (2015). Clinical effect of a positive surgical margin without extraprostatic extension after robot-assisted radical prostatectomy. Urol Oncol.

[6] Sammon JD, Trinh QD, Sukumar S, Ravi P, Friedman A, Sun M, Schmitges J, Jeldres C, Jeong W, Mander N, Peabody JO, Karakiewicz PI, Harris M (2013). Risk factors for biochemical recurrence following radical perineal prostatectomy in a large contemporary series: a detailed assessment of margin extent and location. Urol Oncol.

[7] Sooriakumaran P, Haendler L, Nyberg T, Gronberg H, Nilsson A, Carlsson S, Hosseini A, Adding C, Jonsson M, Ploumidis A, Egevad L, Steineck G, Wiklund P (2012). Biochemical recurrence after robot-assisted radical prostatectomy in a European single-centre cohort with a minimum follow-up time of 5 years. Eur Urol.

[8] Simon RM, Howard LE, Freedland SJ, Aronson WJ, Terris MK, Kane CJ, Amling CL, Cooperberg MR, Vidal AC (2016). Adverse pathology and undetectable ultrasensitive prostate-specific antigen after radical prostatectomy: is adjuvant radiation warranted?. BJU Int.

[9] Touijer KA, Mazzola CR, Sjoberg DD, Scardino PT, Eastham JA (2014). Long-term outcomes of patients with lymph node metastasis treated with radical prostatectomy without adjuvant androgen-deprivation therapy. Eur Urol.

[10] Fairey AS, Daneshmand S, Skinner EC, Schuckman A, Cai J, Lieskovsky G (2014). Long-term cancer control after radical prostatectomy and bilateral pelvic lymph node dissection for pT3bN0M0 prostate cancer in the prostate-specific antigen era. Urol Oncol.

[11] Dindo D, Demartines N, Clavien PA (2004). Classification of surgical complications: a new proposal with evaluation in a cohort of 6336 patients and results of a survey. Ann Surg.

[12] Asimakopoulos AD, Annino F, D'Orazio A, Pereira CF, Mugnier C, Hoepffner JL, Piechaud T, Gaston R (2010). Complete periprostatic anatomy preservation during robot-assisted laparoscopic radical prostatectomy (RALP): the new pubovesical complex-sparing technique. Eur Urol.

[13] Amling CL, Bergstralh EJ, Blute ML, Slezak JM, Zincke H (2001). Defining prostate specific antigen progression after radical prostatectomy: what is the most appropriate cut point?. J Urol.

[14] Olde Heuvel J, de Wit-van der Veen BJ, Huizing DMV, van der Poel HG, van Leeuwen PJ, Bhairosing PA, Stokkel MPM, Slump CH. (2020). State-of-the-art Intraoperative Imaging Technologies for Prostate Margin Assessment: A Systematic Review. Eur Urol Focus.

[15] Novara G, Ficarra V, Mocellin S, Ahlering TE, Carroll PR, Graefen M, Guazzoni G, Menon M, Patel VR, Shariat SF, Tewari AK, Van Poppel H, Zattoni F, Montorsi F, Mottrie A, Rosen RC, Wilson TG (2012). Systematic review and meta-analysis of studies reporting oncologic outcome after robot-assisted radical prostatectomy. Eur Urol.

[16] Ficarra V, Novara G, Secco S, D'Elia C, Boscolo-Berto R, Gardiman M, Cavalleri S, Artibani W (2009). Predictors of positive surgical margins after laparoscopic robot assisted radical prostatectomy. J Urol.

[17] Patel VR, Coelho RF, Rocco B, Orvieto M, Sivaraman A, Palmer KJ, Kameh D, Santoro L, Coughlin GD, Liss M, Jeong W, Malcolm J, Stern JM, Sharma S, Zorn KC, Shikanov S, Shalhav AL, Zagaja GP, Ahlering TE, Rha KH, Albala DM, Fabrizio MD, Lee DI, Chauhan S (2011). Positive surgical margins after robotic assisted radical prostatectomy: a multi-institutional study. J Urol.

[18] Badani KK, Kaul S, Menon M (2007). Evolution of robotic radical prostatectomy: assessment after 2766 procedures. Cancer.

[19] Menon M, Bhandari M, Gupta N, Lane Z, Peabody JO, Rogers CG, Sammon J, Siddiqui SA, Diaz M (2010). Biochemical recurrence following robot-assisted radical prostatectomy: analysis of 1384 patients with a median 5-year follow-up. Eur Urol.

[20] Sukumar S, Rogers CG, Trinh QD, Sammon J, Sood A, Stricker H, Peabody JO, Menon M, Diaz-Insua M (2014). Oncological outcomes after robot-assisted radical prostatectomy: long-term follow-up in 4803 patients. BJU Int.

[21] Murphy DG, Kerger M, Crowe H, Peters JS, Costello AJ (2009). Operative details and oncological and functional outcome of robotic-assisted laparoscopic radical prostatectomy: 400 cases with a minimum of 12 months follow-up. Eur Urol.

[22] Suardi N, Ficarra V, Willemsen P, De Wil P, Gallina A, De Naeyer G, Schatteman P, Montorsi F, Carpentier P, Mottrie A (2012). Long-term Biochemical Recurrence Rates After Robot-assisted Radical Prostatectomy: analysis of a Single-center Series of Patients With a Minimum Follow-up of 5 Years. Urology.

[23] Liss MA, Lusch A, Morales B, Beheshti N, Skarecky D, Narula N, Osann K, Ahlering TE (2012). Robot-assisted radical prostatectomy: 5-year oncological and biochemical outcomes. J Urol.

[24] Diaz M, Peabody JO, Kapoor V, Sammon J, Rogers CG, Stricker H, Lane Z, Gupta N, Bhandari M, Menon M (2015). Oncologic outcomes at 10 years following robotic radical prostatectomy. Eur Urol.

[25] Bravi CA, Tin A, Vertosick E, Mazzone E, Martini A, Dell'Oglio P, Stabile A, Gandaglia G, Fossati N, Suardi N, Gallina A, Briganti A, Montorsi F, Vickers A (2019). The Impact of Experience on the Risk of Surgical Margins and Biochemical Recurrence after Robot-Assisted Radical Prostatectomy: A Learning Curve Study. J Urol.

[26] Wirth MP, Froehner M (2010). Radical prostatectomy-only centers: the future in genitourinary surgery?. Eur Urol.

[27] Tewari AK, Patel ND, Leung RA, Yadav R, Vaughan ED, El-Douaihy Y, Tu JJ, Amin MB, Akhtar M, Burns M, Kreaden U, Rubin MA, Takenaka A, Shevchuk MM (2010). Visual cues as a surrogate for tactile feedback during robotic-assisted laparoscopic prostatectomy: posterolateral margin rates in 1340 consecutive patients. BJU Int..

[28] Gilbert SM, Dunn RL, Miller DC, Montgomery JS, Skolarus TA, Weizer AZ, Wood DP, Hollenbeck BK (2017). Functional Outcomes Following Nerve Sparing Prostatectomy Augmented with Seminal Vesicle Sparing Compared to Standard Nerve Sparing Prostatectomy: Results from a Randomized Controlled Trial. J Urol.

[29] Nguyen LN, Head L, Witiuk K, Punjani N, Mallick R, Cnossen S, Fergusson DA, Cagiannos I, Lavallée LT, Morash C, Breau RH (2017). The Risks and Benefits of Cavernous Neurovascular Bundle Sparing during Radical Prostatectomy: A Systematic Review and Meta-Analysis. J Urol.

[30] Wang X, Wu Y, Guo J, Chen H, Weng X, Liu X (2019). Oncological safety of intrafascial nerve-sparing radical prostatectomy compared with conventional process: a pooled review and meta-regression analysis based on available studies. BMC Urol.

